# Ethyl 4-(4-chloro­anilino)-1-(4-chloro­phen­yl)-2-methyl-5-oxo-2,5-di­hydro-1*H*-pyrrole-2-carboxyl­ate

**DOI:** 10.1107/S1600536813030560

**Published:** 2013-12-03

**Authors:** Mehmet Akkurt, Shaaban K. Mohamed, Mahmoud A.A. Elremaily, Francisco Santoyo-Gonzalez, Mustafa R. Albayati

**Affiliations:** a Department of Physics, Faculty of Sciences, Erciyes University, 38039 Kayseri, Turkey; b Chemistry and Environmental Division, Manchester Metropolitan University, Manchester M1 5GD, England; c Chemistry Department, Faculty of Science, Minia University, 61519 El-Minia, Egypt; d Chemistry Department, Faculty of Science, Sohag University, 82524 Sohag, Egypt; e Department of Organic Chemistry, Faculty of Science, Institute of Biotechnology, Granada University, Granada, E-18071, Spain; f Kirkuk University, College of Science, Department of Chemistry, Kirkuk, Iraq

## Abstract

In the title compound, C_20_H_18_Cl_2_N_2_O_3_, the dihedral angles between the central 2,5-di­hydro-1*H*-pyrrole ring and the phenyl rings are 74.87 (9) and 29.09 (9)°. Intra­molecular N—H⋯O and C—H⋯O hydrogen bonds occur. In the crystal, pairs of N—H⋯O hydrogen bonds link adjacent mol­ecules into inversion dimers and form an *R*_1_^2^(6)*R*_2_^2^(10)*R*_1_^2^(6) ring motif through C—H⋯O hydrogen bonds.

## Related literature   

For the lower toxicity of the lactam ring in comparison to lactones, see: Dembélé *et al.* (1992 ▶). For the importance of lactams in the synthesis of significant bio-active mol­ecules see: Nay *et al.* (2009 ▶); Galeazzi *et al.* (1996 ▶); Ghelfi *et al.* (1999 ▶); Hanessian *et al.* (1996 ▶). For the pharmacological properties of di­hydro­pyrrolo­nes, see: Bergmann & Gericke (1990 ▶); Moody & Young (1994 ▶); Nilsson *et al.* (1990 ▶). For a similar structure, see: Akkurt *et al.* (2013 ▶). For hydrogen-bond motifs, see: Bernstein *et al.* (1995 ▶).
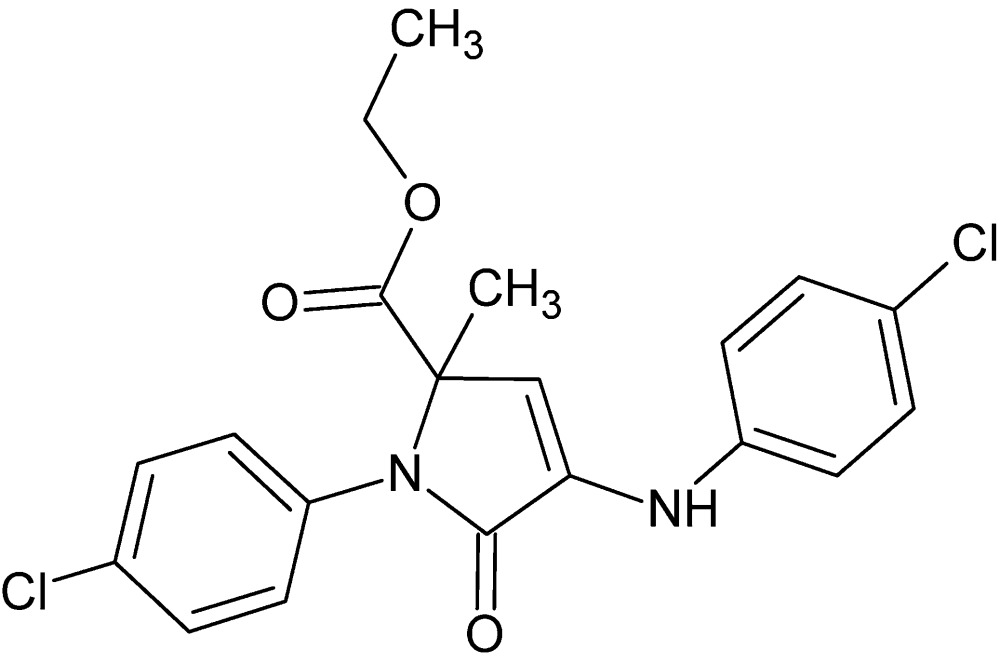


## Experimental   

### 

#### Crystal data  

C_20_H_18_Cl_2_N_2_O_3_
*M* *_r_* = 405.26
Triclinic, 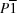

*a* = 5.8319 (3) Å
*b* = 12.3759 (6) Å
*c* = 13.5707 (6) Å
α = 86.484 (2)°
β = 80.098 (2)°
γ = 78.671 (2)°
*V* = 945.69 (8) Å^3^
*Z* = 2
Mo *K*α radiation
μ = 0.37 mm^−1^
*T* = 100 K
0.48 × 0.08 × 0.03 mm

#### Data collection  

Bruker APEXII CCD diffractometer
Absorption correction: numerical (*SADABS*; Bruker, 2005 ▶) *T*_min_ = 0.967, *T*_max_ = 0.990
15209 measured reflections
5316 independent reflections
4054 reflections with *I* > 2σ(*I*)
*R*_int_ = 0.034

#### Refinement  

*R*[*F* ^2^ > 2σ(*F* ^2^)] = 0.045
*wR*(*F* ^2^) = 0.107
*S* = 1.02
5316 reflections
250 parameters
H atoms treated by a mixture of independent and constrained refinement
Δρ_max_ = 0.42 e Å^−3^
Δρ_min_ = −0.26 e Å^−3^

### 

Data collection: *APEX2* (Bruker, 2007 ▶); cell refinement: *SAINT* (Bruker, 2007 ▶); data reduction: *SAINT* (Bruker, 2007 ▶); program(s) used to solve structure: *SIR97* (Altomare *et al.*, 1999) ▶; program(s) used to refine structure: *SHELXL97* (Sheldrick, 2008 ▶); molecular graphics: *ORTEP-3 for Windows* (Farrugia, 2012 ▶); software used to prepare material for publication: *WinGX* (Farrugia, 2012 ▶) and *PLATON* (Spek, 2009 ▶).

## Supplementary Material

Crystal structure: contains datablock(s) global, I. DOI: 10.1107/S1600536813030560/sj5367sup1.cif

Structure factors: contains datablock(s) I. DOI: 10.1107/S1600536813030560/sj5367Isup2.hkl

Supporting information file. DOI: 10.1107/S1600536813030560/sj5367Isup3.cml

970696

Additional supporting information:  crystallographic information; 3D view; checkCIF report

Additional supporting information:  crystallographic information; 3D view; checkCIF report

## Figures and Tables

**Table 1:** Hydrogen-bond geometry (Å, °)

*D*—H⋯*A*	*D*—H	H⋯*A*	*D*⋯*A*	*D*—H⋯*A*
N2—H2*N*⋯O3	0.82 (2)	2.471 (19)	2.8187 (19)	107.0 (15)
N2—H2*N*⋯O3^i^	0.82 (2)	2.12 (2)	2.9158 (19)	164.9 (18)
C12—H12⋯O2^ii^	0.95	2.34	3.285 (2)	176
C13—H13⋯O2	0.95	2.41	3.142 (2)	134
C14—H14*A*⋯O2^iii^	0.98	2.58	3.431 (2)	146
C16—H16⋯O3^i^	0.95	2.53	3.308 (2)	139

Symmetry codes: (i) 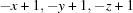; (ii) 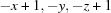; (iii) 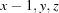.

**Fig. 1.**
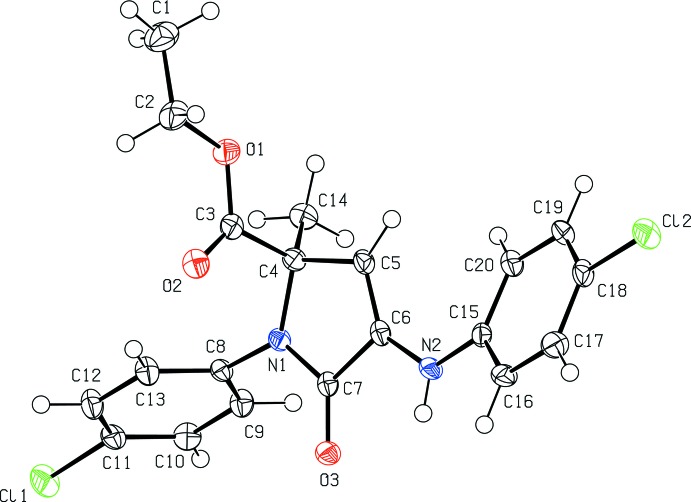
The molecular structure of the title compound with the atom numbering scheme. Displacement ellipsoids for non-H atoms are drawn at the 50% probability level.

**Fig. 2.**
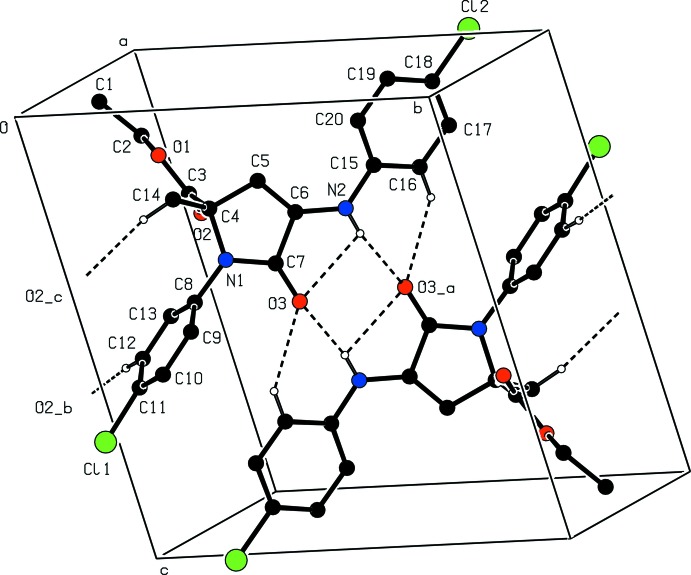
A view of the hydrogen bonding (dotted lines) of the title compound in the unit cell. H atoms not involved in H bonding are omitted for clarity. [Symmetry codes: (a) -x, -y, 1 - z; (b) x, 1 + y, z; (c) 2 - x, 1 - y, -z].

## supplementary crystallographic information

### 1. Comment

Lactam compounds or 2-pyrrolidinones are the aza analogues of lactones. Lactams have received relatively little attention in spite of the fact that they are potentially more effective in a pharmaceutical sense, due to the lower toxicity of the lactam ring with respect to that of the lactone (Dembélé *et al.*, 1992). A number of substances based on the γ-lactam structure have been found in an array of natural products and act as advanced intermediates for the synthesis of many biologically important compounds such as antibiotic and anticancer agents (Nay *et al.*, 2009; Galeazzi *et al.*, 1996; Ghelfi *et al.*, 1999; Hanessian *et al*., 1996). Also, lactams themselves exhibit interesting biological and pharmacological properties, such as psychotropic, antihypertensive and antimuscarinic activity (Bergmann & Gericke 1990; Moody & Young 1994; Nilsson *et al.*, 1990). Based on such facts, and as an extension of our work on the production γ- lactams, we report in this study the synthesis and crystal structure of another dihydro-pyrrolone derivative.

The central 2,5-dihydro-1*H*-pyrrole ring (N1/C4–C7) of the title compound (I), (Fig. 1) makes dihedral angles of 74.87 (9) and 29.09 (9)° with the two phenyl rings (C8···C13 and C15···C20), respectively. All bond lengths and bond angles are normal and are similar to those found in a related compound (Akkurt *et al.*, 2013).

In the crystal, pairs of adjacent molecules are linked through intermolecular N—H···O and C—H···O hydrogen bonds (Table 1), forming an inversion dimer with an *R*^2^_1_(6)*R*^2^_2_(10)*R*^2^_1_(6) ring motif (Bernstein *et al.*, 1995; Fig. 2). In the crystal structure, π-π and C—H···π interactions are not observed.

### 2. Experimental

A mixture of 254 mg (2 mmol) of 4-chloroaniline and 232 mg (2 mmol) of ethyl pyruvate was taken in presence of 8 mol % of Fe_3_O_4_ nanoparticles in 15 ml ethanol/water (*v/v*) and was irradiated in a microwave for 30 minutes. The reaction progress was monitored by TLC. After completion of the reaction, the precipitated solid was filtered off, washed with water and recrystallized from ethanol. Single crystals of the title compound were obtained *via* slow evaporation of an ethanolic solution at room temperature.

### 3. Refinement

The C-bound H-atoms were positioned geometrically [C—H = 0.95, 0.98 and 0.99 Å for aromatic, methyl and methylene H, respectively], and refined by using a riding model, with *U*_iso_(H) = *xU*_eq_(C), where *x* = 1.5 for methyl H, and *x* = 1.2 for the other H atoms. The N-bound H-atom was located from a difference Fourier map and refined freely.

### Crystal data

**Table:**

C_20_H_18_Cl_2_N_2_O_3_	*Z* = 2
*M**_r_* = 405.26	*F*(000) = 420
Triclinic, *P*1	*D*_x_ = 1.423 Mg m^−^^3^
Hall symbol: -P 1	Mo *K*α radiation, λ = 0.71073 Å
*a* = 5.8319 (3) Å	Cell parameters from 6329 reflections
*b* = 12.3759 (6) Å	θ = 2.3–30.1°
*c* = 13.5707 (6) Å	µ = 0.37 mm^−^^1^
α = 86.484 (2)°	*T* = 100 K
β = 80.098 (2)°	Prisms, colourless
γ = 78.671 (2)°	0.48 × 0.08 × 0.03 mm
*V* = 945.69 (8) Å^3^	

### Data collection

**Table:**

Bruker APEXII CCD diffractometer	5316 independent reflections
Radiation source: sealed tube	4054 reflections with *I* > 2σ(*I*)
Graphite monochromator	*R*_int_ = 0.034
φ and ω scans	θ_max_ = 30.5°, θ_min_ = 2.2°
Absorption correction: numerical (*SADABS*; Bruker, 2005)	*h* = −8→8
*T*_min_ = 0.967, *T*_max_ = 0.990	*k* = −17→16
15209 measured reflections	*l* = −19→19

### Refinement

**Table:**

Refinement on *F*^2^	0 restraints
Least-squares matrix: full	H atoms treated by a mixture of independent and constrained refinement
*R*[*F*^2^ > 2σ(*F*^2^)] = 0.045	W = 1/[Σ^2^(*F*O^2^) + (0.044*P*)^2^ + 0.5765*P*] where *P* = (*F*_O_^2^ + 2*F*_C_^2^)/3
*wR*(*F*^2^) = 0.107	(Δ/σ)_max_ = 0.001
*S* = 1.02	Δρ_max_ = 0.42 e Å^−^^3^
5316 reflections	Δρ_min_ = −0.26 e Å^−^^3^
250 parameters	

### Special details

**Table:**

Geometry. Bond distances, angles *etc*. have been calculated using the rounded fractional coordinates. All su's are estimated from the variances of the (full) variance-covariance matrix. The cell e.s.d.'s are taken into account in the estimation of distances, angles and torsion angles
Refinement. Refinement on *F*^2^ for ALL reflections except those flagged by the user for potential systematic errors. Weighted *R*-factors *wR* and all goodnesses of fit *S* are based on *F*^2^, conventional *R*-factors *R* are based on *F*, with *F* set to zero for negative *F*^2^. The observed criterion of *F*^2^ > σ(*F*^2^) is used only for calculating -*R*-factor-obs *etc*. and is not relevant to the choice of reflections for refinement. *R*-factors based on *F*^2^ are statistically about twice as large as those based on *F*, and *R*-factors based on ALL data will be even larger.

### Fractional atomic coordinates and isotropic or equivalent isotropic displacement parameters (Å^2^)

**Table:**

	*x*	*y*	*z*	*U*_iso_*/*U*_eq_	
Cl1	−0.31063 (8)	0.07225 (4)	0.69233 (3)	0.0308 (1)	
Cl2	1.25672 (8)	0.72550 (4)	0.02289 (3)	0.0295 (1)	
O1	0.5731 (2)	0.12044 (10)	0.17897 (9)	0.0241 (3)	
O2	0.6786 (2)	0.14299 (10)	0.32697 (9)	0.0236 (3)	
O3	0.3146 (2)	0.43138 (9)	0.48510 (8)	0.0202 (3)	
N1	0.2726 (2)	0.30149 (11)	0.37842 (10)	0.0170 (3)	
N2	0.5923 (3)	0.51847 (11)	0.32002 (11)	0.0189 (4)	
C1	0.7355 (4)	−0.03397 (17)	0.07524 (16)	0.0357 (6)	
C2	0.7738 (3)	0.02748 (15)	0.16086 (15)	0.0289 (5)	
C3	0.5515 (3)	0.17059 (13)	0.26519 (12)	0.0190 (4)	
C4	0.3517 (3)	0.27458 (13)	0.27306 (12)	0.0184 (4)	
C5	0.4781 (3)	0.36851 (13)	0.23366 (12)	0.0189 (4)	
C6	0.4865 (3)	0.43147 (13)	0.30866 (12)	0.0170 (4)	
C7	0.3497 (3)	0.39131 (13)	0.40231 (12)	0.0160 (4)	
C8	0.1309 (3)	0.24248 (13)	0.45108 (11)	0.0163 (4)	
C9	−0.1047 (3)	0.29019 (14)	0.48361 (13)	0.0209 (4)	
C10	−0.2422 (3)	0.23718 (14)	0.55733 (13)	0.0218 (5)	
C11	−0.1409 (3)	0.13768 (14)	0.59758 (12)	0.0201 (5)	
C12	0.0925 (3)	0.08893 (14)	0.56566 (13)	0.0242 (5)	
C13	0.2296 (3)	0.14242 (14)	0.49218 (13)	0.0212 (4)	
C14	0.1474 (3)	0.26393 (15)	0.22100 (13)	0.0234 (5)	
C15	0.7471 (3)	0.56641 (13)	0.24775 (12)	0.0176 (4)	
C16	0.9122 (3)	0.61854 (14)	0.27971 (12)	0.0212 (4)	
C17	1.0685 (3)	0.66771 (14)	0.21109 (13)	0.0237 (5)	
C18	1.0587 (3)	0.66457 (13)	0.10993 (13)	0.0215 (5)	
C19	0.8948 (3)	0.61546 (14)	0.07666 (13)	0.0238 (5)	
C20	0.7376 (3)	0.56599 (14)	0.14567 (13)	0.0222 (5)	
H1A	0.59060	−0.06430	0.09440	0.0540*	
H1B	0.87100	−0.09420	0.05820	0.0540*	
H1C	0.71990	0.01640	0.01710	0.0540*	
H2A	0.78020	−0.02140	0.22130	0.0350*	
H2B	0.92480	0.05470	0.14400	0.0350*	
H2N	0.591 (3)	0.5340 (16)	0.3776 (15)	0.017 (5)*	
H5	0.54190	0.38060	0.16550	0.0230*	
H9	−0.17120	0.35890	0.45540	0.0250*	
H10	−0.40350	0.26880	0.57980	0.0260*	
H12	0.15800	0.01990	0.59360	0.0290*	
H13	0.39090	0.11050	0.47010	0.0250*	
H14A	0.07270	0.20340	0.25260	0.0350*	
H14B	0.20690	0.24870	0.15020	0.0350*	
H14C	0.03050	0.33290	0.22650	0.0350*	
H16	0.91750	0.62030	0.34920	0.0250*	
H17	1.18060	0.70300	0.23310	0.0280*	
H19	0.88870	0.61530	0.00720	0.0290*	
H20	0.62410	0.53200	0.12320	0.0270*	

### Atomic displacement parameters (Å^2^)

**Table:**

	*U*^11^	*U*^22^	*U*^33^	*U*^12^	*U*^13^	*U*^23^
Cl1	0.0291 (2)	0.0339 (2)	0.0272 (2)	−0.0135 (2)	0.0079 (2)	0.0061 (2)
Cl2	0.0299 (2)	0.0307 (2)	0.0238 (2)	−0.0092 (2)	0.0092 (2)	0.0045 (2)
O1	0.0237 (6)	0.0222 (6)	0.0228 (6)	−0.0007 (5)	0.0025 (5)	−0.0028 (5)
O2	0.0181 (6)	0.0245 (6)	0.0262 (6)	−0.0013 (5)	−0.0016 (5)	0.0013 (5)
O3	0.0212 (6)	0.0213 (6)	0.0175 (6)	−0.0061 (5)	0.0007 (5)	−0.0008 (4)
N1	0.0167 (6)	0.0174 (6)	0.0153 (6)	−0.0042 (5)	0.0023 (5)	0.0008 (5)
N2	0.0232 (7)	0.0200 (7)	0.0135 (7)	−0.0082 (5)	0.0011 (5)	0.0007 (5)
C1	0.0359 (11)	0.0322 (10)	0.0355 (11)	−0.0059 (8)	0.0063 (9)	−0.0096 (8)
C2	0.0246 (9)	0.0249 (9)	0.0311 (10)	0.0019 (7)	0.0063 (7)	−0.0046 (7)
C3	0.0163 (7)	0.0185 (8)	0.0205 (8)	−0.0057 (6)	0.0035 (6)	0.0012 (6)
C4	0.0185 (7)	0.0194 (8)	0.0161 (7)	−0.0049 (6)	0.0018 (6)	0.0002 (6)
C5	0.0206 (8)	0.0175 (8)	0.0167 (7)	−0.0042 (6)	0.0016 (6)	0.0031 (6)
C6	0.0142 (7)	0.0167 (7)	0.0181 (8)	−0.0015 (6)	−0.0006 (6)	0.0039 (6)
C7	0.0126 (7)	0.0156 (7)	0.0184 (7)	−0.0010 (5)	−0.0012 (6)	0.0021 (6)
C8	0.0159 (7)	0.0180 (7)	0.0145 (7)	−0.0052 (6)	0.0009 (6)	0.0007 (6)
C9	0.0171 (7)	0.0196 (8)	0.0244 (8)	−0.0018 (6)	−0.0015 (6)	0.0020 (6)
C10	0.0135 (7)	0.0252 (9)	0.0247 (8)	−0.0024 (6)	0.0012 (6)	−0.0011 (7)
C11	0.0195 (8)	0.0230 (8)	0.0172 (8)	−0.0082 (6)	0.0027 (6)	0.0013 (6)
C12	0.0234 (8)	0.0207 (8)	0.0249 (9)	−0.0020 (7)	0.0013 (7)	0.0064 (7)
C13	0.0151 (7)	0.0216 (8)	0.0232 (8)	−0.0007 (6)	0.0024 (6)	0.0039 (6)
C14	0.0208 (8)	0.0267 (9)	0.0222 (8)	−0.0041 (7)	−0.0029 (7)	−0.0010 (7)
C15	0.0192 (7)	0.0133 (7)	0.0178 (7)	−0.0016 (6)	0.0018 (6)	0.0016 (6)
C16	0.0244 (8)	0.0229 (8)	0.0154 (7)	−0.0071 (7)	0.0023 (6)	−0.0015 (6)
C17	0.0232 (8)	0.0247 (9)	0.0230 (8)	−0.0083 (7)	0.0017 (7)	−0.0016 (7)
C18	0.0222 (8)	0.0175 (8)	0.0209 (8)	−0.0032 (6)	0.0053 (6)	0.0040 (6)
C19	0.0330 (9)	0.0212 (8)	0.0151 (8)	−0.0048 (7)	0.0002 (7)	0.0037 (6)
C20	0.0272 (9)	0.0199 (8)	0.0199 (8)	−0.0073 (7)	−0.0034 (7)	0.0033 (6)

### Geometric parameters (Å, º)

**Table:**

Cl1—C11	1.7431 (17)	C15—C20	1.396 (2)
Cl2—C18	1.7496 (18)	C15—C16	1.398 (2)
O1—C2	1.470 (2)	C16—C17	1.387 (2)
O1—C3	1.333 (2)	C17—C18	1.387 (2)
O2—C3	1.203 (2)	C18—C19	1.377 (3)
O3—C7	1.2245 (19)	C19—C20	1.395 (2)
N1—C4	1.463 (2)	C1—H1A	0.9800
N1—C7	1.354 (2)	C1—H1B	0.9800
N1—C8	1.434 (2)	C1—H1C	0.9800
N2—C6	1.370 (2)	C2—H2A	0.9900
N2—C15	1.401 (2)	C2—H2B	0.9900
N2—H2N	0.82 (2)	C5—H5	0.9500
C1—C2	1.498 (3)	C9—H9	0.9500
C3—C4	1.553 (2)	C10—H10	0.9500
C4—C5	1.518 (2)	C12—H12	0.9500
C4—C14	1.516 (3)	C13—H13	0.9500
C5—C6	1.332 (2)	C14—H14A	0.9800
C6—C7	1.493 (2)	C14—H14B	0.9800
C8—C9	1.391 (2)	C14—H14C	0.9800
C8—C13	1.386 (2)	C16—H16	0.9500
C9—C10	1.387 (2)	C17—H17	0.9500
C10—C11	1.381 (2)	C19—H19	0.9500
C11—C12	1.382 (3)	C20—H20	0.9500
C12—C13	1.387 (2)		
			
C2—O1—C3	115.02 (13)	Cl2—C18—C17	119.29 (13)
C4—N1—C7	112.06 (13)	C17—C18—C19	121.37 (16)
C4—N1—C8	126.06 (13)	C18—C19—C20	119.60 (16)
C7—N1—C8	121.88 (13)	C15—C20—C19	120.01 (16)
C6—N2—C15	127.47 (14)	C2—C1—H1A	109.00
C15—N2—H2N	115.2 (13)	C2—C1—H1B	109.00
C6—N2—H2N	115.5 (13)	C2—C1—H1C	109.00
O1—C2—C1	107.00 (15)	H1A—C1—H1B	109.00
O1—C3—C4	111.43 (14)	H1A—C1—H1C	109.00
O1—C3—O2	124.67 (15)	H1B—C1—H1C	109.00
O2—C3—C4	123.77 (15)	O1—C2—H2A	110.00
N1—C4—C5	101.95 (12)	O1—C2—H2B	110.00
N1—C4—C3	109.18 (13)	C1—C2—H2A	110.00
C3—C4—C14	113.41 (14)	C1—C2—H2B	110.00
C5—C4—C14	114.95 (14)	H2A—C2—H2B	109.00
C3—C4—C5	104.40 (14)	C4—C5—H5	125.00
N1—C4—C14	112.07 (14)	C6—C5—H5	125.00
C4—C5—C6	110.04 (14)	C8—C9—H9	120.00
N2—C6—C5	136.07 (16)	C10—C9—H9	120.00
N2—C6—C7	115.09 (14)	C9—C10—H10	121.00
C5—C6—C7	108.81 (15)	C11—C10—H10	120.00
O3—C7—N1	126.42 (15)	C11—C12—H12	121.00
N1—C7—C6	106.87 (13)	C13—C12—H12	120.00
O3—C7—C6	126.70 (15)	C8—C13—H13	120.00
N1—C8—C13	120.72 (15)	C12—C13—H13	120.00
N1—C8—C9	118.79 (14)	C4—C14—H14A	109.00
C9—C8—C13	120.41 (15)	C4—C14—H14B	109.00
C8—C9—C10	119.87 (16)	C4—C14—H14C	109.00
C9—C10—C11	118.95 (16)	H14A—C14—H14B	109.00
C10—C11—C12	121.86 (16)	H14A—C14—H14C	109.00
Cl1—C11—C12	118.95 (13)	H14B—C14—H14C	109.00
Cl1—C11—C10	119.19 (14)	C15—C16—H16	120.00
C11—C12—C13	118.99 (16)	C17—C16—H16	120.00
C8—C13—C12	119.91 (16)	C16—C17—H17	120.00
C16—C15—C20	119.27 (15)	C18—C17—H17	121.00
N2—C15—C16	118.52 (15)	C18—C19—H19	120.00
N2—C15—C20	122.19 (16)	C20—C19—H19	120.00
C15—C16—C17	120.71 (15)	C15—C20—H20	120.00
C16—C17—C18	119.03 (16)	C19—C20—H20	120.00
Cl2—C18—C19	119.34 (13)		
			
C3—O1—C2—C1	166.64 (15)	C3—C4—C5—C6	−108.42 (16)
C2—O1—C3—O2	−1.9 (2)	N1—C4—C5—C6	5.23 (18)
C2—O1—C3—C4	174.18 (13)	C4—C5—C6—C7	−4.8 (2)
C7—N1—C8—C13	−103.74 (19)	C4—C5—C6—N2	172.91 (19)
C7—N1—C4—C5	−3.72 (17)	N2—C6—C7—O3	3.8 (3)
C8—N1—C4—C5	176.48 (14)	C5—C6—C7—N1	2.39 (19)
C7—N1—C4—C14	−127.16 (15)	N2—C6—C7—N1	−175.86 (14)
C8—N1—C4—C14	53.0 (2)	C5—C6—C7—O3	−177.94 (17)
C4—N1—C8—C9	−107.33 (18)	N1—C8—C13—C12	177.09 (15)
C7—N1—C4—C3	106.33 (15)	N1—C8—C9—C10	−176.92 (15)
C8—N1—C4—C3	−73.47 (19)	C13—C8—C9—C10	−0.3 (3)
C4—N1—C7—C6	1.14 (18)	C9—C8—C13—C12	0.5 (3)
C4—N1—C7—O3	−178.53 (16)	C8—C9—C10—C11	0.4 (3)
C8—N1—C7—O3	1.3 (3)	C9—C10—C11—C12	−0.8 (3)
C4—N1—C8—C13	76.0 (2)	C9—C10—C11—Cl1	178.48 (13)
C8—N1—C7—C6	−179.05 (14)	Cl1—C11—C12—C13	−178.25 (13)
C7—N1—C8—C9	72.9 (2)	C10—C11—C12—C13	1.0 (3)
C15—N2—C6—C7	173.96 (16)	C11—C12—C13—C8	−0.9 (3)
C6—N2—C15—C16	−152.75 (18)	N2—C15—C16—C17	−179.68 (16)
C15—N2—C6—C5	−3.7 (3)	C20—C15—C16—C17	−1.2 (3)
C6—N2—C15—C20	28.8 (3)	N2—C15—C20—C19	179.60 (16)
O1—C3—C4—N1	158.36 (13)	C16—C15—C20—C19	1.2 (3)
O2—C3—C4—C14	−151.28 (17)	C15—C16—C17—C18	0.1 (3)
O1—C3—C4—C5	−93.24 (16)	C16—C17—C18—Cl2	−179.53 (13)
O2—C3—C4—C5	82.87 (19)	C16—C17—C18—C19	1.1 (3)
O2—C3—C4—N1	−25.5 (2)	Cl2—C18—C19—C20	179.51 (13)
O1—C3—C4—C14	32.62 (19)	C17—C18—C19—C20	−1.2 (3)
C14—C4—C5—C6	126.70 (16)	C18—C19—C20—C15	0.0 (3)

### Hydrogen-bond geometry (Å, º)

**Table:**

*D*—H···*A*	*D*—H	H···*A*	*D*···*A*	*D*—H···*A*
N2—H2*N*···O3	0.82 (2)	2.471 (19)	2.8187 (19)	107.0 (15)
N2—H2*N*···O3^i^	0.82 (2)	2.12 (2)	2.9158 (19)	164.9 (18)
C12—H12···O2^ii^	0.95	2.34	3.285 (2)	176
C13—H13···O2	0.95	2.41	3.142 (2)	134
C14—H14*A*···O2^iii^	0.98	2.58	3.431 (2)	146
C16—H16···O3^i^	0.95	2.53	3.308 (2)	139

Symmetry codes: (i) −*x*+1, −*y*+1, −*z*+1; (ii) −*x*+1, −*y*, −*z*+1; (iii) *x*−1, *y*, *z*.
